# Clade I or clade II? Targeting essential viral genes to differentiate monkeypox virus clades by multiplex real-time PCR

**DOI:** 10.3389/fpubh.2025.1626030

**Published:** 2025-08-01

**Authors:** William S. Probert, Alex Espinosa, Jill K. Hacker

**Affiliations:** Viral and Rickettsial Disease Laboratory, Center for Laboratory Sciences, California Department of Public Health, Richmond, CA, United States

**Keywords:** mpox, orthopoxvirus, multiplex polymerase chain reaction, essential genes, disease outbreaks, public health surveillance

## Abstract

The increasing incidence and global spread of mpox have prompted the World Health Organization to twice declare a Public Health Emergency of International Concern. Sustained human-to-human transmission, largely through sexual contact, and waning population immunity to smallpox have accelerated monkeypox virus evolution and driven the emergence of variants that can adversely affect the performance of existing molecular diagnostic tests. To minimize the risk of PCR target drop out and better detect and monitor emerging monkeypox virus variants, we have developed and validated a multiplex real-time PCR (MpoxEG4-plex rPCR) targeting highly conserved and essential orthopoxvirus genes for the detection of four analytes: orthopoxviruses, monkeypox virus, clade I monkeypox virus, and clade II monkeypox virus. The assay limit of detection was ≤ 9 genome copies per reaction, and the clinical accuracy, sensitivity, and specificity were > 96% for each analyte. The new assay was implemented to help confirm the first case of clade I mpox in the United States. The MpoxEG4-plex rPCR offers an accurate, informative, and reliable molecular diagnostic test for identifying cases and tracking case contacts in support of public health efforts to prevent and control the spread of mpox.

## Introduction

The global emergence of mpox has prompted the World Health Organization to issue two declarations of Public Health Emergency of International Concern in response to outbreaks originating in African countries where mpox is endemic (1, 2). From 2022–2024, over 124,000 mpox cases and 272 deaths were reported by 128 countries (3). The initial outbreak, caused by a new monkeypox virus (MPXV) variant, clade IIb lineage B.1, was recognized in May 2022 with over 100,000 cases recorded worldwide and remains ongoing. In September 2023, a second outbreak caused by a new MPXV subclade, designated Ib, was detected in the Democratic Republic of Congo before spreading to neighboring African countries. Travel associated clade Ib cases have been reported by several countries outside of Africa, highlighting the potential for global spread and the need for heightened laboratory surveillance and updated molecular diagnostic tests for mpox.

MPXV is an enveloped, double-stranded DNA virus belonging to the genus *Orthopoxvirus* within the family *Poxviridae*. MPXV, along with other orthopoxviruses (OPXV) including variola (smallpox), vaccinia, camelpox, cowpox, Akhmeta, and borealpox, cause human disease characterized by the presence of localized or generalized vesiculopustular rash or lesions (4). Mpox is endemic to parts of Africa and is caused by two genetically and epidemiologically distinct clades, I and II, which are further divided into subclades a and b. Clade I MPXV is endemic to some central African countries including the Democratic Republic of Congo, Central African Republic, Republic of the Congo, and Gabon, whereas clade II is indigenous to some Western African countries including Nigeria, Ghana, Côte d’Ivoire, Liberia, and Sierra Leone. Both clades are endemic to Cameroon. Mpox caused by clade I has a higher case fatality rate (10.6%) than clade II (3.6%), however, more recent estimates indicate rates of 1–2% for both clades (5, 6). Historically, cases and outbreaks of mpox were due to zoonotic spillover events, with limited secondary transmission. More recently, larger outbreaks of mpox have been fueled by community transmission, particularly through sexual contact with sex workers and among men who have sex with men (7, 8). Selective pressures from sustained human-to-human transmission are believed to be driving accelerated MPXV evolution, leading to more transmissible strains such as clade IIb lineage B.1 and clade Ib (9, 10).

The MPXV genome is approximately 197 kbp in length and contains 193 open reading frames (ORFs) (11). The central region (approximately nucleotides 33,000 to 135,000) is highly conserved among poxviruses and contains essential genes that encode proteins involved in virus replication, gene transcription, and virion morphogenesis, whereas the terminal regions are more variable and contain mostly nonessential genes coding for proteins having roles in host adaptation, pathogenesis, and immune response modulation. At the left and right ends of the genome are identical inverted terminal repetitions (ITRs) of approximately 6.4 kbp. The terminal regions of poxvirus genomes, particularly the ITRs, are susceptible to genetic rearrangement, which can contribute to virus evolution and host adaptation through gene expansion and subsequent gene loss (12, 13). Similar gene duplication and gene deletion events have been described for MPXV, including the 2022 clade IIb and 2023 clade Ib outbreak strains (14–20). Nucleotide substitutions also contribute to MPXV evolution. Although MPXV has a slow rate of mutation relative to RNA viruses, the observation that recent outbreak strains have a higher-than-expected number of nucleotide substitutions supports sustained communicable spread and ongoing host adaptation (10, 16). Indeed, many of the observed nucleotide substitutions were indicative of host-mediated, antiviral apolipoprotein B mRNA editing catalytic polypeptide-like 3 (APOBEC3) cytosine deaminase activity (9, 16).

PCR is the gold standard for laboratory confirmation of mpox (21). Several real-time PCR (rPCR) assays have been developed for detection of mpox and have been designed to provide genus-, species-, clade-, or even subclade-specific information (22–27). However, the emergence of new variants can adversely impact the performance of PCR assays through genetic alterations in sequences targeted for primer or probe binding. This prospect is particularly concerning for PCR assays that target sequences within variable terminal regions and ITRs of the MPXV genome. PCR failures caused by deletions of MPXV species- and clade-specific targets located in the ITRs have been reported for clade Ib strains and a small subset of clade IIb lineage B.1 strains (20, 28). To circumvent this problem and improve the accuracy of PCR for mpox detection, we have developed and validated a quadplex rPCR assay (MpoxEG4-plex rPCR) targeting essential genes in the central conserved region of the MPXV genome to provide genus-, species-, and clade-specific information. This new assay enabled the rapid detection and confirmation of the first case of travel-associated clade I mpox in the United States (29).

## Materials and methods

### DNA and monkeypox viruses

DNA used in this study are listed in Supplementary Table 1. Inactivated MPXV clade Ia strain 2003-ROC-358 and clade IIa strain US_2003 were kindly provided by the Poxvirus & Rabies Branch of the Centers for Disease Control and Prevention (CDC). OPXV genome copies were determined by qPCR using the CDC’s Food and Drug Administration (FDA) 510(k) cleared non-variola *Orthopoxvirus* (NVO) rPCR assay and MPXV synthetic DNA PCR standard (National Institute of Standards and Technology, Gaithersburg, MD) (30).

### Clinical and contrived specimens

Remnant nucleic acids from 61 lesion swab specimens collected in viral or universal transport media were used in this study. The panel included 30 MPXV-negative, 30 clade IIb MPXV-positive, and 1 clade Ib MPXV-positive specimens. Additionally, 30 clade Ia MPXV specimens were contrived by spiking 30 MPXV-negative lesion swab specimens with inactivated MPXV strain 2003-ROC-358 at concentrations ranging from 20,000 to 200,000,000 genome copies per 200 μL. The clinical specimens were submitted to the California Department of Public Health (CDPH) for diagnostic testing and surveillance purposes and were considered exempt from human subject regulations by the California Health and Human Services Agency Committee for the Protection of Human Subjects (Project #2025–058).

### Nucleic acid extraction

Specimens were inactivated by combining 200 μL of each specimen with 200 μL of AL buffer (QIAGEN, Germantown, MD) and 20 μL of protease (QIAGEN) and incubating the mixture at 56°C for 15 min. Nucleic acids were extracted and purified using the NucliSENS easyMAG instrument (bioMérieux, Durham, NC) with an output volume of 100 μL. This procedure has been approved for use with the FDA 510(k) cleared NVO rPCR assay. The nucleic acids were tested immediately or stored at −80°C for retrospective testing.

### Mpox essential genes 4-plex real-time PCR assay (MpoxEG4-plex rPCR)

We assessed the suitability of 90 essential MPXV genes as targets for rPCR assay design (31). Essential gene ORFs and their genomic location were identified and mapped for clade I MPXV strain Zaire-96-I-16 (GenBank reference sequence NC_003310.1) using the coordinates provided by Shchelkunov et al. (11). ORF-by-ORF megablast searches and multiple sequence alignments of representative *Orthopoxvirus* complete genomes were performed using the National Center for Biotechnology Information’s (NCBI) nucleotide collection (nr/nt) database and bioinformatics tools. Regions of ORFs displaying significant sequence divergence at the MPXV species and clade levels were identified visually and recorded as possible candidates for rPCR design. Candidate regions were further refined by filtered megablast searches of the NCBI nr/nt database with only MPXV complete genomes selected as the search set to evaluate target region inclusivity or with MPXV sequences excluded from the search to assess target region exclusivity. Only candidate regions returning search results with 100% target inclusivity and off-target exclusivity were considered for rPCR design. The limited availability of suitable clade I MPXV candidate regions necessitated the selection of a single nucleotide polymorphism (SNP) for the clade I analyte target. The NCBI Primer-Blast tool was used for the design of OPXV-, MPXV- and clade-specific rPCR assays.

Three sets of primers and four differentially labeled fluorogenic probes were used in the MpoxEG4-plex rPCR. Table 1 provides the oligonucleotide sequences, modifications, concentrations, and MPXV genomic coordinates for the primer and probes. The fluorogenic probes were synthesized with either a minor groove binder (Life Technologies Corporation, Carlsbad, CA) or BHQplus (Biosearch Technologies, Novato, CA) modification. The 25 μL reaction consisted of the oligonucleotide primer and probes, 1X PerfeCTa Multiplex qPCR Supermix (Quantabio, Beverly, MA), and 5 μL of nucleic acid extract. Amplification and fluorescence detection were performed on the QuantStudio 5 DX real-time PCR system (Thermo Fisher Scientific, Waltham, MA) using the following parameters: 95°C for 8 min, 40 cycles of 95°C for 3 s and 63°C for 30 s.

**Table 1:** Mpox essential genes 4-plex real-time PCR oligonucleotide primers and probes.

Assay analyte	Gene Target	Oligonucleotide name	Oligonucleotide sequence and modifications*	NCBI Nucleotide sequence coordinates	Assay oligonucleotide concentration
Clade I Monkeypox virus and pan-orthopoxvirus	OPG125	109330F	TGAACGTTTCAAAGTCGAACGAAG	NC_003310.1:109330–109353	400 nM
		109457R	TCCGTTTGATGTGGAAGATACAT	NC_003310.1:109435–109457	400 nM
		MPXV-CI_P	VIC-ACCGTTACCGTAATTT-MGBNFQ	NC_003310.1:109392–109407	200 nM
		PanOPX_P	CFR610-AGTCTTAAACTRTCCGATTCA-BHQ2plus	NC_003310.1:109409–109429	200 nM
Clade II Monkeypox virus	OPG130	111514F	TCAACGCTGGAAGGAGTGTA	ON563414.3:111514–111533	800 nM
		111627R	CTCCTGTACTAAAACCACGTCA	ON563414.3:111606–111627	800 nM
		MPXV-CII_P	FAM-TGGTTGTCTACAGATACT-MGBNFQ	ON563414.3:111548–111565	200 nM
Monkeypox virus	OPG084	64966F	ATCACTAGGAAAACCCGCACAT	ON563414.3:64966–65987	250 nM
		65044R	ACATTCAAAGCTTATTGCATACTCACTA	ON563414.3:65017–65044	250 nM
		MPXV-Gen_P	Q670-TTGCTTCGACATTAGGTT -BHQ2plus	ON563414.3:64991–65008	70 nM

*Oligonucleotide modifications: VIC, CFR610 (Cal Fluor Red 610), FAM, and Q670 (Quasar 670) are fluorescent dyes. MGBNFQ (Minor Groove Binder Non-Fluorescent Quencher) and BHQ2plus (Black Hole Quencher 2 plus) are non-fluorescent acceptor dyes.

A rPCR assay targeting human RNaseP was performed concomitantly as an endogenous control to ensure proper specimen collection and sample integrity.

### Comparator rPCR assays

The FDA-cleared NVO real-time PCR assay targeting E9L was used as the comparator rPCR test for the detection of OPXV (30). For the detection of MPXV and clade Ia or clade II MPXV, a laboratory developed triplex rPCR validated by the CDPH for diagnostic use served as the comparator test. The MPXV triplex rPCR assay targets the G2R and C3L regions described by Li et al. (32) with minor modifications to improve inclusivity and enable multiplexing (Supplementary material). Only the MPXV generic analyte will be detected for clade Ib strains (C3L deletion) with this assay (27).

### Determination of MpoxEG4-plex rPCR performance characteristics

To assess MPXV and OPXV primer and probe sequence inclusivity, 6,904 MPXV complete genome sequences (> 196,000 bp) with < 5% undetermined nucleotides and parsed by analyte (MPXV, clade I, or clade II) were downloaded from the GISAID Epipox database (accessed February 26, 2025) and interrogated using the python script PCR_strainer, and the PCR simulation program Thermonucleotide BLAST (33, 34). Sequences identified by PCR_strainer as having undetermined nucleotides in a target sequence were excluded, providing 6,886 MPXV, 260 clade I, 6,607 clade II, and 6,879 OPXV sequences for inclusivity analyses. Among the clade I sequences, 245 were assigned to subclade Ia and 15 to subclade Ib, and for the clade II sequences, 6 were assigned to subclade IIa and 6,601 to subclade IIb. Inclusivity results were documented as the number of sequences with 0, 1, 2, or ≥ 3 mismatches for each primer-probe set. In addition, 95 MPXV clade IIb A.2.2 complete genome sequences representing the proposed G.1 lineage were downloaded from the NCBI MPXV data hub (accessed July 5, 2025), and the MpoxEG4-plex rPCR primer and probe sequences were mapped to these genomes to assess inclusivity using Geneious Prime 2022.0.2 (Biomatters, Auckland, New Zealand). Finally, 292 non-MPXV orthopoxvirus sequences were available through the NCBI core nucleotide database and assessed for OPXV primer and probe sequence inclusivity using the NCBI multiple sequence alignment tool (accessed February 27, 2025). This cohort consisted of 1 Abatino, 6 Akhmeta, 1 borealpox, 6 buffalopox, 10 camelpox, 97 cowpox, 5 ectromelia, 3 horsepox, 1 rabbitpox, 1 raccoonpox, 1 skunkpox, 1 taterapox, 81 vaccinia, 77 variola, and 1 volepox complete genome sequences.

In silico exclusivity of the primer and probe sequences was assessed through individual blastn searches against the NCBI reference genome sequences for *Homo sapiens* and 33 microorganisms as recommended by the FDA (35). DNA from organisms sharing ≥ 80% sequence homology with a primer or probe sequence and from 10 additional OPXV were tested with the MpoxEG4-plex assay for cross-reactivity (Supplementary Table 1).

Assay analytical sensitivity or limit of detection (LOD) was determined for each analyte using inactivated and quantified MPXV strains 2003-ROC-358 (clade I) and US_2003 (clade II) spiked into pooled MPXV-negative lesion swab matrix and processed through the entire test workflow. A preliminary LOD was established for decreasing three-fold serial concentrations ranging from 243 to 0.33 genome copies per reaction tested in replicates of three. A second run was then performed at the preliminary LOD and two immediately higher concentrations in replicates of seven. The data from these two runs were combined and the LOD for each analyte defined as the concentration for which all 10 replicates were detected.

Accuracy, sensitivity, and specificity were determined for each analyte by comparison of test results from the MpoxEG4-plex rPCR with those from the comparator tests for the panel of 61 clinical and 30 contrived specimens.

## Results

### Design and development of the MpoxEG4-plex rPCR assay

**Figure 1:**
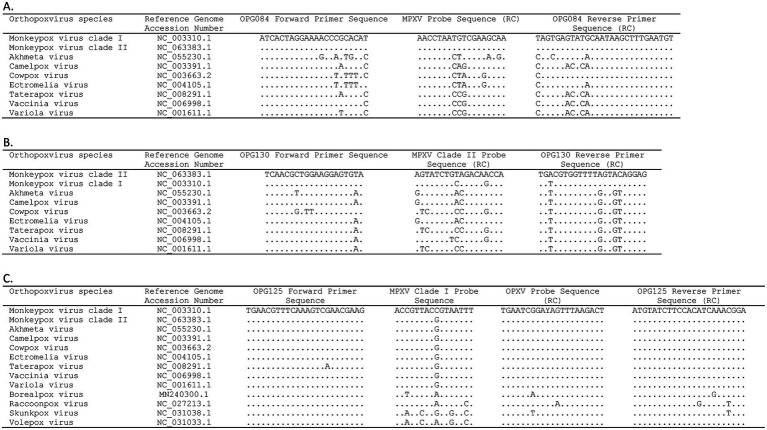
Alignment of mpox essential genes 4-plex real-time PCR **(A)** OPG084, **(B)** OPG130, and **(C)** OPG125 primer and probe sequences with orthopoxvirus reference sequences. Dots indicate identical nucleotide for that position. RC, reverse complement.

Three gene targets, OPG084, OPG125, and OPG130, were selected for assay design based on systematic ORF-by-ORF alignment and comparison of 90 essential gene sequences from representative OPXV genomes (Table 1) (31). The OPG084 and OPG130 primer and probe sets detect MPXV species and clade II MPXV strains, respectively, whereas the OPG125 primer and duplex probe set differentially detects clade I MPXV and OPXV. In silico analyses of complete genome sequences revealed that the primer and probe sets are highly inclusive with perfect matches to 99.4, 100, 99.9, and 99.8% of the MPXV, clade I, clade II, and OPXV target sequences, respectively (Table 2). The OPG084 primer and probe sequences are highly conserved among MPXV and broadly divergent among other OPXV (Table 2; Figure 1A). Similarly, the OPG130 primer and probe sequences are highly conserved among clade II MPXV strains but display a significant number of nucleotide mismatches with other OPXV species (Table 2; Figure 1B). The ability of the OPG130 target to distinguish clade II MPXV strains from clade I strains, however, is largely dependent on two nucleotide mismatches between the probe sequence and clade I sequences (Figure 1B). The OPG125 primer sequences and OPXV probe sequence are widely conserved among both Old and New World OPXV (Table 2; Figure 1C). This target utilizes a second probe sequence that is highly conserved among clade I MPXV strains and allows differentiation from clade II MPXV and other OPXV based largely on a SNP (Table 2; Figure 1C). The differentiation of the clade I and clade II analytes were facilitated by the design and use of allelic discrimination probes.

**Table 2:** *In silico* analyses of mpox essential genes 4-plex real-time PCR primer and probe sequences for inclusivity.

Primer-probe set (Gene target)	Number of complete genome sequences				
	Total queried	Perfect sequence match	1 nucleotide mismatch	2 nucleotide mismatch	3 nucleotide mismatch
Monkeypox virus (OPG084)	6,981	6,936	45	0	0
Clade I Monkeypox virus (OPG125)	260	260	0	0	0
Clade II Monkeypox virus (OPG130)	6,702	6,698	4	0	0
Orthopoxvirus (OPG125)	7,266*	7,249	14**	2***	1****

*Includes complete genome sequences from monkeypox, cowpox, camelpox, Akhmeta, ectromelia, vaccinia, variola, taterapox, buffalopox, Abatino, rabbitpox, horsepox, borealpox, volepox, skunkpox, and raccoonpox viruses.

**Monkeypox (9), cowpox (3), taterapox (1), vaccinia (1).

***Borealpox and skunkpox.

****Raccoonpox.

### MpoxEG4-plex rPCR assay performance characteristics

Assay cross-reactivity was first assessed by in silico analyses of primer and probe sequences for ≥ 80% homology with the human genome sequence and the genome sequences of 33 microorganisms, as specified in the FDA’s Emergency Use Authorization Template for Mpox (35). Significant sequence homology was observed with 24 genome sequences. Assay cross-reactivity with representative DNA from these 23 organisms (variola virus excluded) and 9 additional OPXV strains was not detected (Supplementary Table 1). As expected, DNA representing 13 OPXV strains were detected for the OPXV analyte but not for the MPXV analytes (Table 3).

**Table 3:** Mpox essential genes 4-plex real-time PCR cross-reactivity with *Orthopoxvirus* DNA.

*Orthopoxvirus*	Strain	MPXV Species analyte Ct	Clade I MPXV analyte Ct	Clade II MPXV analyte Ct	OPXV analyte Ct
Vaccinia	MVA	Not detected	Not detected	Not detected	21.3
Ectromelia	MOS	Not detected	Not detected	Not detected	21.7
Camelpox*	Negev 2016	Not detected	Not detected	Not detected	18.8
Cowpox clade 1	Fin_2000	Not detected	Not detected	Not detected	19.3
Cowpox clade 3	EP-4	Not detected	Not detected	Not detected	17.7
Cowpox clade 4	Norway 1994	Not detected	Not detected	Not detected	20
Cowpox clade 5*	No-H2	Not detected	Not detected	Not detected	21.5
Akhmeta	2013–88	Not detected	Not detected	Not detected	23.6
Taterapox	DAH68	Not detected	Not detected	Not detected	17.6
Raccoonpox	MD61	Not detected	Not detected	Not detected	27.7
Volepox	CA85	Not detected	Not detected	Not detected	22.2
Skunkpox	WA78	Not detected	Not detected	Not detected	27.8
Borealpox	AK2015	Not detected	Not detected	Not detected	23.2

MPXV, monkeypox virus; Ct, cycle threshold value; OPXV, orthopoxvirus.

*Synthetic DNA concatemer of target sequences.

The assay LOD was determined for each analyte using quantified inactivated clade I and clade II viruses spiked into pooled MPXV-negative lesion swab matrix and processed through the entire assay workflow. LOD determinations of 1 copy and 3 copies per reaction were established for the MPXV analyte with the clade I and clade II viruses, respectively, 3 copies per reaction for the OPXV analyte with both viruses, 3 copies per reaction for the clade I MPXV analyte, and 9 copies per reaction for the clade II MPXV analyte (Tables 4, 5).

**Table 4:** Mpox essential genes 4-plex real-time PCR limit of detection analysis for clade I monkeypox virus.

Genome copies per reaction	Monkeypox virus analyte		Clade I Monkeypox virus analyte		Clade II Monkeypox virus analyte		Orthopoxvirus analyte	
	Number of replicates detected	Mean Ct value*	Number of replicates detected	Mean Ct value	Number of replicates detected	Mean Ct value	Number of replicates detected	Mean Ct value
243	3/3	27 (0.45)	3/3	28.5 (0.91)	0/3	ND	3/3	26.9 (0.86)
81	3/3	29.1 (0.28)	3/3	31 (0.22)	0/3	ND	3/3	29.1 (0.34)
27	3/3	30.4 (0.10)	3/3	32.1 (0.14)	0/3	ND	3/3	30.3 (0.12)
9	10/10	32.6 (0.69)	10/10	34.6 (0.75)	0/10	ND	10/10	33.1 (0.94)
3	10/10	34.4 (0.62)	10/10	35.7 (0.37)	0/10	ND	10/10	34.3 (0.62)
1	10/10	35.6 (1.07)	9/10	37.1 (0.97)	0/10	ND	9/10	35.7 (1.02)
0.33	0/3	ND	0/3	ND	0/3	ND	1/3	35

ND, not detected.

*Ct, cycle threshold; standard deviation in parentheses.

**Table 5:** Mpox essential genes 4-plex real-time PCR limit of detection analysis for clade II monkeypox virus.

Genome copies per reaction	Monkeypox virus analyte		Clade I Monkeypox virus analyte		Clade II Monkeypox virus analyte		Orthopoxvirus analyte	
	No of replicates detected	Mean Ct value*	Number of replicates detected	Mean Ct value	Number of replicates Detected	Mean Ct value	Number of replicates detected	Mean Ct value
243	3/3	28.8 (0.18)	0/3	ND	3/3	30.7 (0.10)	3/3	28.9 (0.20)
81	3/3	29.9 (0.12)	0/3	ND	3/3	31.7 (0.17)	3/3	30 (0.21)
27	3/3	31.6 (0.11)	0/3	ND	3/3	32.9 (0.19)	3/3	31.2 (0.35)
9	10/10	33.1 (0.43)	0/10	ND	10/10	34.6 (0.65)	10/10	33.2 (0.49)
3	10/10	35.1 (1.03)	0/10	ND	9/10	36.2 (0.92)	10/10	35.2 (0.92)
1	7/10	36 (0.71)	0/10	ND	7/10	37.1 (1.26)	8/10	36 (1.38)
0.33	2/3	36.3 (0.86)	0/3	ND	1/3	38.2	1/3	36.6

ND, not detected.

*Ct, cycle threshold; standard deviation in parentheses.

Clinical agreement was determined by retrospectively testing 61 clinical specimens (1 clade Ib MPXV positive, 30 clade IIb MPXV positives, and 30 MPXV negatives) and 30 contrived clade Ia MPXV positive specimens and comparing test results with the FDA-cleared NVO assay or the laboratory developed triplex assay for the detection of MPXV species, clade Ia MPXV, and clade II MPXV (Supplementary Table 2). The accuracy, sensitivity, and specificity of the MpoxEG4-plex assay were all 100% for the OPXV, MPXV, and clade I MPXV analytes, and 98.9, 96.7, and 100% for the clade II MPXV analyte, respectively. Only a single discordant analyte result was observed; clinical specimen 54 was positive for the clade II MPXV analyte (Ct = 34) with the comparator test but negative for the MpoxEG4-plex rPCR clade II analyte. The OPXV (Ct = 37.1) and MPXV (Ct = 36.9) analytes were detected for this specimen with the MpoxEG4-plex rPCR assay. As expected, the clade Ib specimen (clinical specimen 61) was detected for the OPXV, MPXV, and clade I analytes with the MpoxEG4-plex rPCR assay and the non-variola OPXV and MPXV analytes with the comparator tests. With the triplex MPXV rPCR comparator test, this specimen was positive for the generic MPXV analyte but negative for the clade Ia (C3L target) and clade II analytes. Whole genome sequencing (WGS) confirmed this specimen as clade Ib (36).

## Discussion

We have developed and validated a multiplex rPCR assay targeting three essential genes for the detection of OPXV genus, MPXV species, and MPXV clades I and II. All three gene targets, OPG125, OPG084, and OPG130, are located within the central conserved region of the MPXV genome and encode proteins having an essential role in either viral replication or morphogenesis (31). OPG125 (MPXV ORF E13L) encodes a scaffold protein that is highly conserved among poxviruses and considered essential for morphogenesis of vaccina virus (homolog D13L) (37). The OPG125 target is amplified using primer sequences broadly conserved among OPXV; the amplicon is detected with two differentially labeled probes: one recognizing an OPXV-specific sequence and the other targeting a MPXV clade I-specific SNP in a separate region of the amplicon. The MPXV species target, OPG084 (MPXV ORF I8R), encodes a DNA and RNA helicase, which is required for early gene transcription in vaccinia virus (38). For clade II MPXV detection, the assay targets OPG130 (MPXV ORF A5L), an immunodominant 39-kDa virion core protein that is required for vaccinia virus maturation (39).

Validation of the MpoxEG4-plex assay established an analytical sensitivity of ≤ 9 genome copies per reaction for all four analytes and confirmed that the targets were highly specific for the intended analyte by both in silico analyses and cross-reactivity testing. Assay accuracy, sensitivity, and specificity were > 96% for all analytes with only a single discordant analyte result observed relative to the comparator tests: a clade II analyte result was falsely negative for one specimen, likely due to the MPXV nucleic acid concentration being at the lower limit of analyte detection. Overall, the performance characteristics of the MpoxEG4-plex rPCR assay were equivalent to the comparator tests but with added inclusivity for clade Ib. Notably, implementation of the MpoxEG4-plex rPCR assay was instrumental in recognizing the first clade I infection in a traveler returning to the United States from Eastern Africa (36).

Numerous PCR targets have been explored for the detection of OPXV and MPXV (22–27). However, many of the MPXV species- and clade-specific assays target genes that are nonessential and located within the variable terminal regions of the genome. While sequence diversity within these regions may offer species- and clade-specific targets, they also may be subject to gene loss through genetic rearrangement. Two commonly used PCR targets for MPXV species and clade II identification are located within OPG002 (MPXV ORFs J2L and J2R; often referred to as G2R, the variola homolog), a gene present in both ITRs (32). False negative PCR results attributed to a 600-bp deletion within both copies of OPG002 and consequently, the loss of MPXV species and clade II targets have been recently reported (28). Fortunately, the appearance of strains with this deletion seems to have been geographically isolated and transient. In contrast, a more sustained and diagnostically consequential deletion emerged with subclade Ib (20). These strains possess a 1,114-bp deletion encompassing OPG032 (MPXV ORF D14L; vaccinia homolog C3L) that adversely impacts the performance of a commonly used clade I-specific PCR (27). These two examples of gene deletion illustrate the potential risk associated with targeting the terminal variable regions of the MPXV genome for the design of PCR assays, particularly given the propensity of poxviruses to evolve and adapt to new hosts through gene expansion and subsequent deletion.

Nucleotide substitutions also play an important role in poxvirus evolution and host adaptation. Poxviruses have an estimated substitution rate of 1 to 2 nucleotides per genome per year (40). The substitution rate for clade IIb MPXV, however, increased 6 to 12-fold in the last 10 years, likely because of sustained human-to-human transmission (16). Substitutions observed with the emergent 2022 outbreak clade IIb variant B.1 and 2023 outbreak clade Ib strains are predominantly driven by host-mediated, antiviral APOBEC3 cytosine deaminase activity (nucleotide replacements GA > AA and TC > TT) (16, 18). Remarkably, the 2022 lineage B.1 outbreak sequence differed by 46 nucleotide substitutions from a 2018 lineage A.1 reference sequence with nearly 90% of the replacements likely associated with APOBEC3 activity (16). This accelerated evolution can impact the performance of MPXV PCR assays. PCR primer sequence mismatches with clade IIb lineage B.1 sequences have been shown to reduce the analytical sensitivity of MPXV PCR assays (41, 42). Moreover, several recent studies using in silico analyses of MPXV genome sequence compilations have documented a significant occurrence of primer and probes sequence mismatches that may impact the performance of many published PCR assays (42–45). Conversely, the MpoxEG4-plex rPCR primer and probe sequences are highly inclusive with perfect matches of > 99.4% to the intended OPXV/MPXV genome sequences (Table 2).

The appearance of APOBEC3 mutations in clade IIb, lineage B.1 genomes follow a nonrandom genome distribution including several mutational hotspots, and a higher-than-expected frequency of synonymous mutations and mutations in noncoding regions and inverted repeats (9, 46). Sustained human-to-human transmission likely will continue to drive MPXV diversity through the accumulation of APOBEC3 nucleotide substitutions that do not significantly impact viral fitness. Future APOBEC3 mutations are predicted to manifest in TC dinucleotide hotspots present in the ITRs, large coding sequences (> 1.8 kbp), and transcriptionally active genes (47, 48). The selection of PCR targets located within the central conserved region of the MPXV genome, and the presumed functional and structural constraints of essential genes minimizes the risk of MpoxEG4-plex rPCR primer and probe sequence mismatches as a consequence of future APOBEC3 mutations. Moreover, the multianalyte design of the MpoxEG4-plex assay will assist in the rapid recognition of discordant results should a MPXV variant emerge with target sequence alterations. Nevertheless, assay inclusivity should be monitored for drift by periodic queries of MPXV genome databases for primer and probe sequence mismatches. For example, the MpoxEG4-plex rPCR primer and probes sequences were reassessed with the emergence of newly described clade IIb A.2.2.1 (proposed lineage G.1) strains associated with an outbreak in Sierra Leone and found to match this lineage perfectly (49).

A limitation of our study was the paucity of clade I positive specimens and strains available for testing. Clade I MPXV, but not clade II, is a select agent in the United States, and its transfer, possession, and use are subject to federal regulation. To address the lack of clade I clinical specimens, we contrived specimens using known concentrations of a single inactivated clade Ia strain. In addition, we received and tested a suspected clade I MPXV specimen during assay validation that was confirmed as clade Ib by WGS (36). While only two clade I strains were evaluated with the MpoxEG4-plex assay, in silico analyses supported excellent primer-probe binding inclusivity for clade I genome sequences. Nonetheless, assay robustness would benefit from additional testing using a diverse panel of MPXV strains. Another limitation was the evaluation of only a single specimen type, lesion swabs. Whereas lesion material is considered the optimal specimen for MPXV PCR, validation of additional specimen types such as rectal and oropharyngeal swabs would be useful for testing patients lacking an obvious rash or lesion and may contribute to case finding and public health intervention efforts (e.g., contact tracing) (21).

With sustained human-to-human transmission, MPXV will continue to evolve at an accelerated pace and new variants will likely emerge that may present diagnostic challenges for existing molecular tests (50). The multianalyte configuration of the MpoxEG4-plex assay and the selection of highly conserved essential gene targets will reduce the risk of false-negative test results due to emerging MPXV variants. Moreover, the ability of the MpoxEG4-plex assay to provide clade level information will facilitate tracking clade distributions and introductions into nonendemic regions. Finally, the new assay promises to provide rapid, accurate, and informative test results to help guide the public health response to prevent and control the spread of mpox.

## Data Availability

The original contributions presented in the study are included in the article/Supplementary material, further inquiries can be directed to the corresponding author.
