# Supplementary material for: Clade I or clade II? Targeting essential viral genes to differentiate monkeypox virus clades by multiplex real-time PCR
Source: Front Public Health. 2025 Aug 1;13:1626030. doi: 10.3389/fpubh.2025.1626030 (PMC12354367; doi:10.3389/fpubh.2025.1626030)
Supplement: Supplementary file 1 [file Table_1.docx]

**Clade I or clade II? Targeting essential viral genes to differentiate monkeypox virus clades by multiplex real-time PCR**

**Supplementary Materials:**

**Monkeypox virus triplex real-time PCR assay**

The MPXV triplex rPCR assay targets the G2R (G2R_G = Monkeypox Generic and G2R_WA = Clade II) and C3L (Clade I) regions described by Li et al. (1) with minor modifications to the G2R_G primers and the G2R_WA probe to improve inclusivity and enable multiplexing: G2R_G forward primer – GGAAARTGTAAAGACAACGAATACAG, G2R_G reverse primer – GCTATCACATAATCTGRAAGCGTA, and G2R_WA probe – ACCCGTCGTAACCAGCAATACATT. For multiplexing, the G2R_G, C3L, and G2R_WA probes were labeled with FAM, VIC, and ABY, respectively (Life Technologies Corporation, Carlsbad, CA). The multiplex reaction consisted of 1X Premix Ex Taq (Probe qPCR) master mix (Takara Bio USA, San Jose, CA), G2R_G primers at 400 nM each, G2R_G probe at 100 nM, C3L primers at 400 nM each, C3L probe at 100 nM, G2R_WA primers at 200 nM, and G2R_WA probe at 100 nM. Amplification and fluorescence detection were performed on the ABI 7500 FAST DX Sequence Detection System (Thermo Fisher Scientific, Waltham, MA) using the fast mode and the following parameters: 95°C for 1 min, and 45 cycles of 95°C for 3 s and 60°C for 30 s. The assay was validated by California Department of Public Health for diagnostic use in compliance with the Clinical Laboratory Improvement Amendments of 1988 as a laboratory developed test. It has not been cleared by the Food and Drug Administration.

1. Li Y, Zhao H, Wilkins K, Hughes C, Damon IK. Real-time PCR assays for the specific detection of monkeypox virus West African and Congo Basin strain DNA. J Virol Methods. (2010) 169:223-7. doi: 10.1016/j.jviromet.2010.07.012.

Table S1. DNA samples used for mpox essential genes 4-plex real-time PCR cross reactivity testing

| Organism | Strain | Source | DNA copies tested |
| --- | --- | --- | --- |
| Akhmeta virus | 2013-88 | CDC | 29,000 |
| Borealpox virus | AK2015 | CDC | Not quantified |
| Camelpox virus* | Negev 2016 | IDT | 100,000 |
| Cowpox virus clade 1 | Fin_2000 | CDC | 100,000 |
| Cowpox virus clade 3 | EP-4 | CDC | 78,000 |
| Cowpox virus clade 4 | Norway 1994 | CDC | 100,000 |
| Cowpox virus clade 5* | No-H2 | IDT | 100,000 |
| Ectromelia virus | MOS | CDC | 100,000 |
| Raccoonpox virus | MD61 | CDC | Not quantified |
| Skunkpox virus | WA78 | CDC | Not quantified |
| Taterapox virus | DAH68 | CDC | 100,000 |
| Vaccinia virus | MVA | ATCC | 100,000 |
| Volepox virus | CA85 | CDC | Not quantified |
| *Acinetobacter baumannii* | H72721 | BEI Resources | 1,000,000 |
| *Bacteroides fragilis* | VP1 | ATCC | 1,000,000 |
| *Candida albicans* | 12C | BEI Resources | 1,000,000 |
| *Chlamydia trachomatis* | UW-3/Cx | ATCC | 1,000,000 |
| *Enterococcus faecalis* | TX0104 | BEI Resources | 1,000,000 |
| *Escherichia coli* | CDC 9707 | BEI Resources | 1,000,000 |
| *Lactocaseibacillus rhamnosus* | LMS2-1 | BEI Resources | 1,000,000 |
| *Mycoplasma pneumoniae* | NCTC 10119 | ATCC | 1,000,000 |
| *Neisseria gonorrhoeae* | FA1090 | ATCC | 1,000,000 |
| *Pseudomonas aeruginosa* | PAO1-LAC | ATCC | 1,000,000 |
| *Staphylococcus aureus* | MN8 | BEI Resources | 1,000,000 |
| *Staphylococcus epidermidis* | SK135 | BEI Resources | 1,000,000 |
| *Streptococcus* Group C | 92A06081 (2) | CDPH | 1,000,000 |
| *Streptococcus* Group G | 02S00244 | CDPH | 1,000,000 |
| *Streptococcus mitis* | NCTC 12261 | ATCC | 1,000,000 |
| *Streptococcus pyogenes* | T1 | ATCC | 1,000,000 |
| *Trichomonas vaginalis* | G3 | ATCC | 1,000,000 |
| *Trichophyton rubrum* | M22D000131 | CDPH | 500,000 |
| *Homo sapien* | Female | Promega Corporation | 350,000 |

CDC = Centers for Disease Control and Prevention, IDT = Integrated DNA Technologies, ATCC = American Type Culture Collection, CDPH = California Department of Public Health

*Synthetic DNA concatemer encompassing the mpox essential genes 4-plex real-time PCR target sequences

Table S2. Agreement between the mpox essential genes 4-plex real-time PCR assay and comparator PCR assays

| Specimen | Comparator PCR Tests* | | | | Mpox essential genes 4-plex PCR | | | | Endogenous Control PCR |
| --- | --- | --- | --- | --- | --- | --- | --- | --- | --- |
|  | Non-variola OPXV Ct | Triplex generic MPXV Ct | Triplex clade I MPXV Ct | Triplex clade II MPXV Ct | MPXV  species Ct | Clade I MPXV Ct | Clade II MPXV Ct | OPXV Ct | Human RNaseP Ct |
| Clinical 1 | Not Detected | Not Detected | Not Detected | Not Detected | Not Detected | Not Detected | Not Detected | Not Detected | 32.5 |
| Clinical 2 | Not Detected | Not Detected | Not Detected | Not Detected | Not Detected | Not Detected | Not Detected | Not Detected | 29.7 |
| Clinical 3 | Not Detected | Not Detected | Not Detected | Not Detected | Not Detected | Not Detected | Not Detected | Not Detected | 31.4 |
| Clinical 4 | Not Detected | Not Detected | Not Detected | Not Detected | Not Detected | Not Detected | Not Detected | Not Detected | 29.3 |
| Clinical 5 | Not Detected | Not Detected | Not Detected | Not Detected | Not Detected | Not Detected | Not Detected | Not Detected | 35.3 |
| Clinical 6 | Not Detected | Not Detected | Not Detected | Not Detected | Not Detected | Not Detected | Not Detected | Not Detected | 29.8 |
| Clinical 7 | Not Detected | Not Detected | Not Detected | Not Detected | Not Detected | Not Detected | Not Detected | Not Detected | 23.7 |
| Clinical 8 | Not Detected | Not Detected | Not Detected | Not Detected | Not Detected | Not Detected | Not Detected | Not Detected | 24.7 |
| Clinical 9 | Not Detected | Not Detected | Not Detected | Not Detected | Not Detected | Not Detected | Not Detected | Not Detected | 29.6 |
| Clinical 10 | 17.5 | 17.0 | Not Detected | 15.7 | 16 | Not Detected | 18.1 | 16.2 | 25.8 |
| Clinical 11 | 22.8 | 21.5 | Not Detected | 20.7 | 21.6 | Not Detected | 23.4 | 21.7 | 31.5 |
| Clinical 12 | 23.8 | 21.8 | Not Detected | 20.6 | 23 | Not Detected | 24.8 | 23.2 | 27.6 |
| Clinical 13 | Not Detected | Not Detected | Not Detected | Not Detected | Not Detected | Not Detected | Not Detected | Not Detected | 30.8 |
| Clinical 14 | Not Detected | Not Detected | Not Detected | Not Detected | Not Detected | Not Detected | Not Detected | Not Detected | 28.9 |
| Clinical 15 | Not Detected | Not Detected | Not Detected | Not Detected | Not Detected | Not Detected | Not Detected | Not Detected | 35 |
| Clinical 16 | Not Detected | Not Detected | Not Detected | Not Detected | Not Detected | Not Detected | Not Detected | Not Detected | 31.8 |
| Clinical 17 | Not Detected | Not Detected | Not Detected | Not Detected | Not Detected | Not Detected | Not Detected | Not Detected | 27.6 |
| Clinical 18 | Not Detected | Not Detected | Not Detected | Not Detected | Not Detected | Not Detected | Not Detected | Not Detected | 28.8 |
| Clinical 19 | Not Detected | Not Detected | Not Detected | Not Detected | Not Detected | Not Detected | Not Detected | Not Detected | 25.9 |
| Clinical 20 | Not Detected | Not Detected | Not Detected | Not Detected | Not Detected | Not Detected | Not Detected | Not Detected | 24.7 |
| Clinical 21 | Not Detected | Not Detected | Not Detected | Not Detected | Not Detected | Not Detected | Not Detected | Not Detected | 29.7 |
| Clinical 22 | Not Detected | Not Detected | Not Detected | Not Detected | Not Detected | Not Detected | Not Detected | Not Detected | 29.2 |
| Clinical 23 | Not Detected | Not Detected | Not Detected | Not Detected | Not Detected | Not Detected | Not Detected | Not Detected | 25.6 |
| Clinical 24 | Not Detected | Not Detected | Not Detected | Not Detected | Not Detected | Not Detected | Not Detected | Not Detected | 30.4 |
| Clinical 25 | Not Detected | Not Detected | Not Detected | Not Detected | Not Detected | Not Detected | Not Detected | Not Detected | 27.8 |
| Clinical 26 | Not Detected | Not Detected | Not Detected | Not Detected | Not Detected | Not Detected | Not Detected | Not Detected | 33.7 |
| Clinical 27 | Not Detected | Not Detected | Not Detected | Not Detected | Not Detected | Not Detected | Not Detected | Not Detected | 26.3 |
| Clinical 28 | Not Detected | Not Detected | Not Detected | Not Detected | Not Detected | Not Detected | Not Detected | Not Detected | 26.3 |
| Clinical 29 | Not Detected | Not Detected | Not Detected | Not Detected | Not Detected | Not Detected | Not Detected | Not Detected | 25.9 |
| Clinical 30 | Not Detected | Not Detected | Not Detected | Not Detected | Not Detected | Not Detected | Not Detected | Not Detected | 28.2 |
| Clinical 31 | Not Detected | Not Detected | Not Detected | Not Detected | Not Detected | Not Detected | Not Detected | Not Detected | 33.9 |
| Clinical 32 | 24.2 | 22.3 | Not Detected | 22.3 | 22.6 | Not Detected | 24.9 | 23 | 27.4 |
| Clinical 33 | 27.4 | 25.7 | Not Detected | 25.5 | 25.7 | Not Detected | 27.9 | 26.2 | 35.8 |
| Clinical 34 | 19.8 | 18.5 | Not Detected | 18.4 | 17.9 | Not Detected | 20.5 | 18.6 | 25.4 |
| Clinical 35 | 17.7 | 16.5 | Not Detected | 16.5 | 15.9 | Not Detected | 18.5 | 16.6 | 23.1 |
| Clinical 36 | Not Detected | Not Detected | Not Detected | Not Detected | Not Detected | Not Detected | Not Detected | Not Detected | 29.1 |
| Clinical 37 | 29.8 | 27.8 | Not Detected | 28.1 | 28 | Not Detected | 30.3 | 28.5 | 34 |
| Clinical 38 | Not Detected | Not Detected | Not Detected | Not Detected | Not Detected | Not Detected | Not Detected | Not Detected | 28.5 |
| Clinical 39 | 17.9 | 17.9 | Not Detected | 15.4 | 16.6 | Not Detected | 18.3 | 16.7 | 22.5 |
| Clinical 40 | 20.0 | 18.2 | Not Detected | 17.3 | 18.5 | Not Detected | 20.3 | 18.3 | 20.3 |
| Clinical 41 | 19.9 | 18.6 | Not Detected | 17.7 | 18.9 | Not Detected | 20.9 | 19 | 23.6 |
| Clinical 42 | 18.7 | 17.7 | Not Detected | 16.9 | 17.4 | Not Detected | 19.4 | 17.4 | 27.9 |
| Clinical 43 | 19.7 | 18.3 | Not Detected | 18.0 | 18.5 | Not Detected | 20.4 | 18.5 | 22.4 |
| Clinical 44 | 31.5 | 31.0 | Not Detected | 29.5 | 30.6 | Not Detected | 32.5 | 30.7 | 35.4 |
| Clinical 45 | 16.1 | 15.8 | Not Detected | 15.0 | 14.9 | Not Detected | 17 | 15.2 | 23.9 |
| Clinical 46 | 21.9 | 20.6 | Not Detected | 19.2 | 20.5 | Not Detected | 22.8 | 20.8 | 26.3 |
| Clinical 47 | 18.3 | 17.5 | Not Detected | 17.1 | 17.5 | Not Detected | 19.3 | 17.4 | 24.4 |
| Clinical 48 | 20.6 | 17.9 | Not Detected | 17.2 | 18.9 | Not Detected | 20.9 | 18.9 | 23.8 |
| Clinical 49 | 30.9 | 30.0 | Not Detected | 28.2 | 29.3 | Not Detected | 31.3 | 29.8 | 31.5 |
| Clinical 50 | 30.9 | 30.3 | Not Detected | 28.8 | 30.3 | Not Detected | 32.2 | 30.6 | 36.8 |
| Clinical 51 | 23.8 | 23.3 | Not Detected | 21.7 | 22.5 | Not Detected | 24.3 | 22.8 | 28.4 |
| Clinical 52 | 21.5 | 20.0 | Not Detected | 18.0 | 20.3 | Not Detected | 22.2 | 20.7 | 23.2 |
| Clinical 53 | 18.3 | 17.5 | Not Detected | 16.4 | 17.6 | Not Detected | 19.4 | 17.8 | 32.3 |
| Clinical 54 | 36.5 | 34.4 | Not Detected | 34.0 | 36.9 | Not Detected | Not Detected | 37.1 | 28.5 |
| Clinical 55 | 14.1 | 14.2 | Not Detected | 13.9 | 13.3 | Not Detected | 15.1 | 13.6 | 20.9 |
| Clinical 56 | 29.3 | 26.9 | Not Detected | 24.5 | 28 | Not Detected | 30 | 28.5 | 35.9 |
| Clinical 57 | 27.6 | 25.8 | Not Detected | 23.9 | 26.7 | Not Detected | 28.6 | 27.2 | 32.7 |
| Clinical 58 | 15.2 | 14.3 | Not Detected | 14.0 | 13.7 | Not Detected | 15.8 | 14.3 | 22 |
| Clinical 59 | 20.2 | 18.0 | Not Detected | 16.3 | 19.2 | Not Detected | 21 | 19.3 | 26 |
| Clinical 60 | 17.6 | 16.2 | Not Detected | 15.2 | 16.5 | Not Detected | 18.4 | 16.8 | 26.4 |
| Clinical 61 | 18.1 | 17.8 | Not Detected | Not Detected | 18.7 | 20.3 | Not Detected | 18.7 | 22.6 |
| Contrived 1 | 13.8 | 12.7 | 13.7 | Not Detected | 12.8 | 14.3 | Not Detected | 12.9 | 28 |
| Contrived 2 | 14.0 | 13.0 | 13.8 | Not Detected | 12.9 | 14.1 | Not Detected | 12.9 | 25.6 |
| Contrived 3 | 14.3 | 13.2 | 13.8 | Not Detected | 13.3 | 14.7 | Not Detected | 13.1 | 28.3 |
| Contrived 4 | 15.2 | 14.1 | 14.9 | Not Detected | 13.8 | 15.4 | Not Detected | 14 | 28.2 |
| Contrived 5 | 14.1 | 13.3 | 14.1 | Not Detected | 12.9 | 14.6 | Not Detected | 13.1 | 26.7 |
| Contrived 6 | 11.2 | 10.6 | 11.1 | Not Detected | 8.9 | 11.4 | Not Detected | 9.8 | 25.5 |
| Contrived 7 | 11.5 | 11.1 | 11.6 | Not Detected | 10.4 | 11.5 | Not Detected | 9.6 | 25.5 |
| Contrived 8 | 14.8 | 13.7 | 14.5 | Not Detected | 13.8 | 15.3 | Not Detected | 13.9 | 27.8 |
| Contrived 9 | 15.0 | 14.1 | 15.0 | Not Detected | 13.6 | 13.9 | Not Detected | 12.8 | 29.3 |
| Contrived 10 | 16.1 | 15.3 | 16.1 | Not Detected | 15 | 16.4 | Not Detected | 15.1 | 28.7 |
| Contrived 11 | 17.3 | 16.6 | 16.6 | Not Detected | 16.3 | 17.6 | Not Detected | 16.3 | 23.6 |
| Contrived 12 | 17.1 | 16.2 | 16.4 | Not Detected | 15.6 | 17.2 | Not Detected | 15.7 | 28.5 |
| Contrived 13 | 16.9 | 16.2 | 16.5 | Not Detected | 15.6 | 17.3 | Not Detected | 15.8 | 23.9 |
| Contrived 14 | 16.9 | 16.2 | 16.3 | Not Detected | 15.9 | 17.4 | Not Detected | 16 | 22.7 |
| Contrived 15 | 16.4 | 16.0 | 16.1 | Not Detected | 15.4 | 17 | Not Detected | 15.4 | 22.9 |
| Contrived 16 | 20.5 | 19.3 | 19.6 | Not Detected | 19 | 21 | Not Detected | 19.3 | 22 |
| Contrived 17 | 20.3 | 19.2 | 19.5 | Not Detected | 19 | 20.9 | Not Detected | 19.2 | 24.9 |
| Contrived 18 | 20.0 | 19.3 | 19.8 | Not Detected | 19.3 | 21.2 | Not Detected | 19.3 | 25 |
| Contrived 19 | 20.4 | 19.4 | 19.9 | Not Detected | 19.4 | 21.3 | Not Detected | 19.6 | 24.8 |
| Contrived 20 | 20.3 | 19.3 | 19.6 | Not Detected | 19.2 | 21.2 | Not Detected | 19.5 | 24.3 |
| Contrived 21 | 23.2 | 22.4 | 22.6 | Not Detected | 21.8 | 23.8 | Not Detected | 22.2 | 23.3 |
| Contrived 22 | 23.6 | 22.9 | 23.3 | Not Detected | 22.6 | 24.6 | Not Detected | 23 | 29.2 |
| Contrived 23 | 23.7 | 23.0 | 23.3 | Not Detected | 22.7 | 24.7 | Not Detected | 23 | 24.6 |
| Contrived 24 | 23.7 | 23.0 | 23.2 | Not Detected | 22.5 | 24.4 | Not Detected | 22.8 | 23.6 |
| Contrived 25 | 23.8 | 22.7 | 23.1 | Not Detected | 22.5 | 24.2 | Not Detected | 22.6 | 25.3 |
| Contrived 26 | 27.5 | 26.7 | 27.1 | Not Detected | 26.3 | 28 | Not Detected | 26.6 | 25.6 |
| Contrived 27 | 27.7 | 26.8 | 27.1 | Not Detected | 26.4 | 28.2 | Not Detected | 26.6 | 36.2 |
| Contrived 28 | 27.6 | 27.1 | 27.3 | Not Detected | 26.4 | 28 | Not Detected | 26.4 | 29.7 |
| Contrived 29 | 27.9 | 27.2 | 27.4 | Not Detected | 26.4 | 28 | Not Detected | 26.6 | 25.1 |
| Contrived 30 | 26.1 | 26.4 | 26.7 | Not Detected | 25.5 | 27.3 | Not Detected | 25.8 | 26.2 |

MPXV = Monkeypox virus, OPXV = Orthopoxvirus, Ct = cycle threshold value

*Comparator tests include an FDA-cleared Non-variola *Orthopoxvirus* real-time PCR assay and a laboratory-developed Monkeypox virus triplex real-time PCR assay
